# Quantifying the escalating impact of paramedic transported emergency department visits for opioid-related conditions in Ontario, Canada: A population-based cohort study

**DOI:** 10.1371/journal.pone.0291194

**Published:** 2023-09-08

**Authors:** Ryan P. Strum, Shawn Mondoux, Fabrice I. Mowbray, Paul Miller, Andrew Worster, Richard Ferron, Andrew P. Costa

**Affiliations:** 1 Department of Health Research Methods, Evidence and Impact, McMaster University, Hamilton, Canada; 2 Department of Medicine, Division of Emergency Medicine, McMaster University, Hamilton, Canada; 3 Institute for Health Policy, Management and Evaluation, University of Toronto, Toronto, Canada; 4 College of Nursing, Michigan State University, East Lansing, Michigan, United States of America; 5 Centre for Paramedic Education and Research, Hamilton Health Sciences, Hamilton, Canada; 6 Niagara Emergency Medical Services, Niagara on the Lake, Ontario, Canada; 7 Department of Medicine, McMaster University, Hamilton, Canada; CHU Nantes, FRANCE

## Abstract

**Introduction:**

While overdoses comprise the majority of opioid research, the comprehensive impact of the opioid crisis on emergency departments (EDs) and paramedic services has not been reported. We examined temporal changes in population-adjusted incidence rates of ED visits and paramedic transports due to opioid-related conditions.

**Materials and methods:**

We conducted a population-based cohort study of all ED visits in the National Ambulatory Care Reporting System from January 1, 2009 to December 31, 2019 in Ontario, Canada. We included all patients with a primary diagnosis naming opioids as the underlying cause for the visit, without any other drugs or substances. We clustered geographic regions using Local Health Integration Network boundaries. Descriptive statistics, incidence rate ratios (IRR) and 95% confidence intervals (CIs) were calculated to analyze population-adjusted temporal changes.

**Results:**

Overall, 86,403 ED visits were included in our study. Incidence of opioid-related ED visits increased by 165% in the study timeframe, with paramedic transported patients increasing by 429%. Per 100,000 residents, annual ED visits increased from 40.4 to 97.2, and paramedic transported patients from 12.1 to 67.9. The proportion of opioid-related ED visits transported by paramedics increased from 35.0% to 69.9%. The medical acuity of opioid-related ED visits increased throughout the years (IRR 6.8. 95% CI 5.9–7.7), though the proportion of discharges remained constant (~75%). The largest increases in ED visits and paramedic transports were concentrated to urbanized regions.

**Discussion:**

Opioid-related ED visits and paramedic transports increased substantially between 2009 and 2019. The proportion of ED visits transported by paramedics doubled. Our findings could provide valuable support to health stakeholders in implementing timely strategies aimed at safely reducing opioid-related ED visits. The increased use of paramedics followed by high rates of ED discharge calls for exploration of alternative care models within paramedic systems, such as direct transport to specialized substance abuse centres.

## Introduction

The opioid crisis continues to be a public health challenge for Canada. In recent years more than eight people a day die of opioid-related poisonings in Canada [1, 2]. Despite considerable public health interventions to guard against opioid abuse and overdose, the national rates of mortality and hospital admissions continue to rise [3]. The root cause of Canada’s opioid crisis is complex and dependent on several factors, including increased prescription rates of long-acting oxycodone, prescription of opioids in high doses, and increased potency of illicit opioid drugs, mostly fentanyl and oxycodone [4–6]. This combination has cultivated an international healthcare opioid crisis in the past decade.

Between 2015 and 2017, Ontario reported a sharp increase in hospital visits and admissions related to opioids, representing a new wave of the opioid crisis in the province [7]. Although specific subgroups of opioid-related emergency department (ED) visits, such as overdoses and deaths, have been extensively studied, there is a lack of comprehensive understanding regarding the broader clinical impact of opioids on EDs and the overall health care system, such as mental health disorders due to opioids and opioid withdrawal syndromes. Furthermore, the magnitude of the effect of the opioid crisis on paramedic services, and clinical characteristics of ED transported patients, is unknown. No comprehensive research reports how ED and paramedic service utilization have changed during the opioid crisis in Ontario in the past decade that adjust for population-growth. As the prevalence of opioid disorders and overdoses continue to rise, it is important to quantify the full magnitude of the opioid epidemic impact on the healthcare system. Understanding the complete extent of this crisis on EDs and paramedic services could inform the urgency with which alternative interventions should be implemented to improve patient centred care for people who use opioids.

Our objective was to describe and quantify incidence changes of ED visits and paramedic transported visits due to opioid-related conditions in Ontario, Canada. We examined trends within the cohort for age, acuity, geographic location and year of visit, after adjusting for population growth.

## Materials and methods

### Study design

We conducted a population-based retrospective cohort study using administrative ED records from the National Ambulatory Care Reporting System (NACRS) database. We adhered to the Reporting of Studies Conducted Using Observational Routinely-Collected Health Data (RECORD) statement for the reporting of results [8].

### Population and setting

All patients who were triaged in an Ontario ED between January 1, 2009 to December 31, 2019 were eligible for inclusion. We included all ED visits that had a primary diagnosis of an opioid-related condition as the reason for visit, as assigned by the ED physician. To reduce contamination and isolate visits specific to opioids, main diagnoses that included opioids among other illicit drugs were excluded. This study timeframe represents the most recently available decade prior to the COVID-19 pandemic, reducing any time-specific potential confounders likely to influence health outcomes and health service beyond 2019.

### Data source

We extracted data from the NACRS database, a hospital and community-based ambulatory care administrative database that collects data for every patient’s ED visit at the time of service use in Ontario [9]. NACRS is housed within the Institute for Clinical Evaluative Sciences (ICES), a non-profit, independent corporation that supports the study of health service and outcomes at a population-level in Ontario. Data concerning Ontario’s population as a province and for each region were extracted from the Registered Persons Database (RPDB) at ICES. RPDB is a repository of all persons registered in Ontario under the Ontario Health Insurance Plan (OHIP) and eligible for universal healthcare services. These databases are routinely checked for quality and accuracy [10]. We identified the study population from the NACRS population database; we did not use any data cleaning methods as NACRS data are recorded using structured codification.

### Variables and measurement

We identified the study population using the International Statistical Classification of Diseases and Related Health Problems 10^th^ Revision (ICD-10) codification system, as recorded in the main diagnostic category of the ED visit [11]. S1 Table shows the individual ICD-10 codes used to identify opioid-related ED visits in this study; individual patient-level data were aggregated to examine a complete ED visitation depiction of annual opioid-related visits. Patient age was originally extracted as twenty-two categorical levels due to personal health information privacy restrictions. The Canadian Triage and Acuity Scale (CTAS) was used to operationalize triage of patients at entry to ED, assigned by the ED triage nurse. CTAS is an ordinal scale that ranges from one to five, with a score of one indicating the highest acuity (resuscitation) and five the lowest acuity (non-urgent) [12]. Regionalization of Ontario was determined using the location of the ED within fourteen Local Health Integration Network (LHIN) boundaries. LHIN boundaries divide Ontario into fourteen smaller geographic sections, and are responsible for the planning and funding of hospital services within their geographic region.

### Statistical analysis

We reported descriptive statistics using frequencies and proportions. We calculated incidence rates per 100,000 residents of the population for each year. We computed incidence rate ratios (IRR) with using 95% confidence intervals (CIs) to adjust for population growth as a predictor of ED visitation increases for opioid-related conditions [13]. Data were managed and analyzed in R (v. 3.6) [14]. We reported missing data directly and handled using complete-case analysis.

### Ethics approval

ICES’s collection and use of NACRS secondary ambulatory data is authorized under Section 45 of Ontario’s Personal Health Information Protection Act (PHIPA) as a prescribed entity, which is exempt from review by a Research Ethics Board [15, 16]. The use of the data in this study is authorized under section 45 and approved by ICES’s Privacy and Legal Office.

## Results

Overall, 86,403 ED visits between January 1, 2009 and December 31, 2019 were included in the study; no data were excluded or missing. Table 1 shows annual incidence changes of ED visits due to opioid-related conditions. Over the 11-year study period, total ED visits increased by 165% (8,741), with paramedic transported patients increasing by 429% (7,956). Opioid-related ED visits increased by 24 visits per day (14.5 to 38.5), and paramedic transports increased by 21.8 (5.1 to 26.9) per day. The proportion of ED visits transported by paramedics doubled from 35.0% to 69.9%. Adjusting for population growth, ED visits increased by 56.9 per 100,000 residents, constituting an IRR of 2.4 (95% CI 2.3–2.5). Paramedic transports increased by 53.8 per 100,000 residents, yielding an IRR of 4.8 (95% CI 4.6–5.0). A significant surge was observed in years 2016 and 2017 for both ED visits (IRR 1.2, 95% CI 1.2–1.2; IRR 1.4 95% CI 1.4–1.5 respectively) and paramedic transports (IRR 1.3, 95% CI 1.2–1.3; IRR 1.7, 85% CI 1.7–1.8 respectively).

**Table 1:** Changes of annual opioid-related ED visits and paramedic transports incidence and rate ratios in Ontario, Canada from January 1, 2009 to December 31, 2019.

Year	All ED Visits				Paramedic Transported ED Visits			
	Incidence	Incidence Rate Per 100,000	ED Visits per Day	IRR (95% CI)^a^	Incidence (% of all ED Visits)	Incidence Rate Per 100,000	Transports per Day	IRR (95% CI)^a^
**2009**	5,303	40.4	14.5	-	1,854 (35.0)	14.1	-	-
**2010**	6,070	46.1	16.6	1.1 (1.1–1.2)	2,071 (34.1)	15.7	5.1	1.1 (1.0–1.2)
**2011**	6,344	47.7	17.4	1.0 (1.0–1.2)	2,253 (35.5)	16.9	5.7	1.1 (1.0–1.2)
**2012**	6,181	46.0	16.9	1.0 (1.0–1.2)	2,484 (40.2)	18.5	6.2	1.1 (1.0–1.2)
**2013**	5,649	41.6	15.5	0.9 (0.9–0.9)	2,488 (44.0)	18.3	6.8	1.0 (0.9–1.1)
**2014**	5,717	41.8	15.7	1.0 (1.0–1.1)	2,725 (47.7)	19.9	6.8	1.1 (1.0–1.2)
**2015**	6,247	45.3	17.1	1.1 (1.0–1.1)	3,194 (51.1)	23.2	7.5	1.2 (1.1–1.2)
**2016**	7,525	53.9	20.6	1.2 (1.1–1.2)	4,080 (54.2)	29.2	8.8	1.3 (1.2–1.3)
**2017**	11,148	79.0	30.5	1.5 (1.4–1.5)	7,158 (64.2)	50.7	11.2	1.7 (1.7–1.8)
**2018**	12,175	85.3	33.4	1.1 (1.0–1.1)	8,157 (67.0)	57.1	19.6	1.1 (1.1–1.2)
**2019**	14,044	97.2	38.5	1.1 (1.1–1.2)	9,810 (69.9)	67.9	22.3	1.2 (1.2–1.2)
**Total Increase**	8,741 (165%)	56.8	24.0	2.4 (2.4–2.5)	7,956 (429%)	53.8	26.9	4.8 (4.6–5.0)

**Note**: ED = Emergency Department, IRR = Incident Rate Ratio, CI = Confidence Interval

^a^ Ratio represents comparison of year to previous year.

Table 2 shows the distribution of ED visits based on type of opioid problem over the study years, the largest being poisoning (46% all ED visits, 68% paramedic transported), and mental and behavioural disorders due to opioid withdrawal (28% all ED visits, 14% paramedic transported).

**Table 2:** Proportion of opioid-related ED visits in the National Ambulatory Care Reporting System from 2009 to 2019.

Description of NACRS Codification of Opioid-Related Conditions	Proportion of Total ED Visits (%)	Proportion of Paramedic Transports (%)
**Total mental and behavioural disorders**	54.5	32.1
Mental and behavioural disorders due to use of opioids	0.0	0.0
Mental and behavioural disorders due to use of opioids, acute intoxication	3.3	4.2
Mental and behavioural disorders due to use of opioids, harmful use	12.2	10.6
Mental and behavioural disorders due to use of opioids, dependence syndrome	9.8	2.8
Mental and behavioural disorders due to use of opioids, withdrawal state	27.5	13.6
Mental and behavioural disorders due to use of opioids, withdrawal state with delirium	0.2	0.1
Mental and behavioural disorders due to use of opioids, psychotic disorder	0.7	0.4
Mental and behavioural disorders due to use of opioids, amnesic syndrome	0.0	0.0
Mental and behavioural disorders due to use of opioids, residual and late-onset psychotic disorder	0.0	0.0
Mental and behavioural disorders due to use of opioids, other mental and behavioural disorders	0.2	0.1
Mental and behavioural disorders due to use of opioids, unspecified mental and behavioural disorders	0.5	0.3
**Total Poisoning**	45.5	67.9
Poisoning by narcotic and psychodysleptics	0.0	0.0
Poisoning by opium	0.2	0.3
Poisoning by heroin	7.5	12.7
Poisoning by other opioids	14.0	18.6
Poisoning by morphine	0.4	0.6
Poisoning by hydromorphone	0.8	1.2
Poisoning by oxycodone	0.9	1.3
Poisoning by other opioids, not elsewhere classified	3.4	5.7
Poisoning by methadone	3.4	4.0
Poisoning by other synthetic narcotics	4.0	5.9
Poisoning by fentanyl and derivatives	5.2	8.7
Poisoning by tramadol	0.1	0.1
Poisoning by other synthetic narcotics, not elsewhere classified	0.1	0.2
Poisoning by other and unspecified narcotics	5.5	8.5
**Finding of opiate drug in blood**	0.0	0.0

**Note:** NACRS = National Ambulatory Care Reporting System

**Fig 1:**
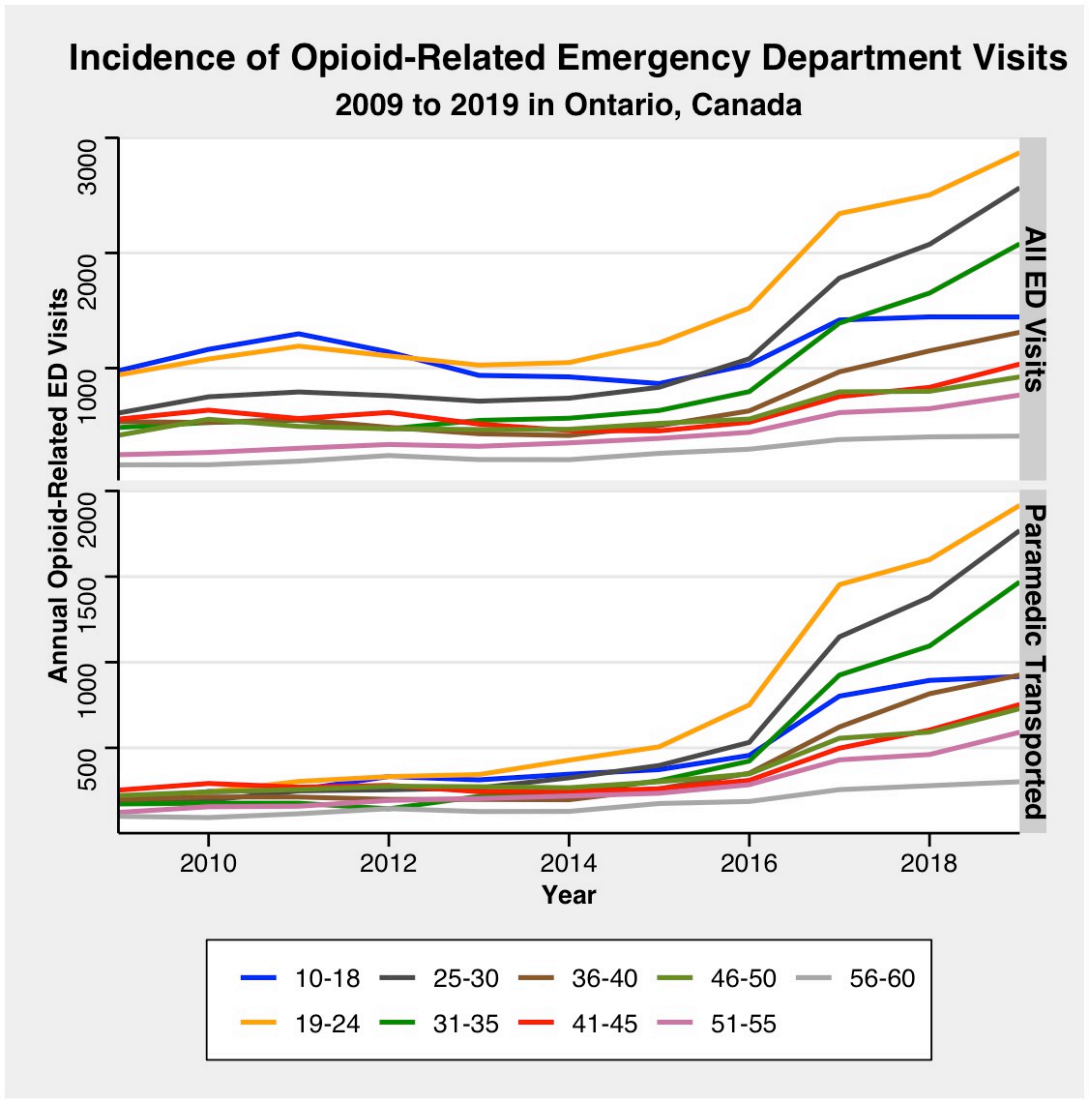
Incidence of opioid-related ED visits and transported by paramedics from 2009 to 2019.

Fig 1 displays surges in opioid-related ED visits across all age groups between 10 and 60 years of age during the study years, specifically during 2016 and 2017. The annual incidence of paramedic transported patients remained relatively constant between 2009 to 2015, but experienced an exponential increase thereafter. Ages between 0 and 9 years, and 71 to 105 years, are not shown as incidence were relatively scant.

Table 3 shows descriptive statistics of ED visits of 2009 and 2019. In these years, the majority of patients shifted from female to male. Increases in the most emergent triage acuity scores were observed (CTAS 1 to 3), while lower acuity scores decreased (CTAS 4 and 5). ED visit disposition remained consistent across these years, with over three quarters of visits discharged without any hospital admission (76.2% in 2009, 75.6% in 2019). The proportion of patients returning to the ED within 30 days increased slightly for all ED visits and those transported by paramedics over the study period (33.1% to 36.3%, 30.2% to 35.3%, respectively).

**Table 3:** Characteristics and rate ratios of opioid-related ED visits between 2009 and 2019 in Ontario, Canada.

	All ED Visits					Paramedic Transported ED Visits				
	2009		2019		Incidence Rate Ratio (95% CI)	2009		2019		Incidence Rate Ratio (95% CI)
	Visits, n (%)	Incident Rate Per 100,000	Visits, n (%)	Incident Rate Per 100,000		Visits, n (%)	Incident Rate Per 100,000	Visits, n (%)	Incident Rate Per 100,000	
**Total**	5,303	40.7	14,044	97.7	2.4 (2.3–2.5)	1,854	14.2	9,810	68.2	4.8 (4.6–5.0)
**Gender**										
Male	2,285 (43.1)	17.6	9,279 (66.1)	64.5	3.7 (3.5–3.9)	1,019 (55.0)	7.8	6,632 (67.6)	46.1	5.9 (5.5–6.3)
Female	3,018 (56.9)	23.2	4,765 (33.9)	33.1	1.4 (1.4–1.5)	835 (45.0)	6.4	3,178 (32.4)	22.1	3.4 (3.2–3.7)
**Triage Acuity**, CTAS										
Resuscitation (1)	246 (4.6)	1.9	1,839 (13.1)	12.8	6.8 (5.9–7.7)	228 (12.3)	1.8	1,657 (16.9)	11.5	6.6 (5.7–7.6)
Emergent (2)	1,493 (28.2)	11.5	6,729 (47.9)	46.8	4.1 (3.9–4.3)	897 (48.4)	6.9	5,456 (55.6)	37.9	5.5 (5.1–5.9)
Urgent (3)	2,065 (38.9)	15.9	4,378 (31.2)	30.4	1.9 (1.8–2.0)	601 (32.4)	4.6	2,486 (25.3)	17.3	3.7 (3.4–4.1)
Less Urgent (4)	1,093 (20.6)	8.4	830 (5.9)	5.8	0.7 (0.6–0.8)	116 (6.3)	0.9	196 (2.0)	1.4	1.5 (1.2–1.9)
Non-Urgent (5)	406 (7.7)	3.1	268 (1.9)	1.9	0.6 (0.5–0.7)	12 (0.6)	0.1	15 (0.2)	0.1	1.1 (0.5–2.4)
**ED Visit Outcome**										
Discharged	4,043 (76.2)	31.1	10,623 (75.2)	73.9	2.4 (2.3–2.5)	1,113 (60.6)	8.6	7,196 (73.4)	50.0	5.9 (5.5–6.2)
Admitted	841 (15.9)	6.5	1,803 (12.8)	12.5	1.9 (1.8–2.1)	570 (30.7)	4.4	1,351 (13.8)	9.4	2.2 (1.9–2.4)
Other	419 (7.9)	3.2	1,618 (11.5)	11.3	3.5 (3.1–3.9)	171 (9.2)	1.3	1,263 (12.9)	8.8	6.7 (5.7–7.9)
**Returned to any ED within 30 days**										
Yes	1,755 (33.1)	13.5	5,099 (36.3)	35.5	2.6 (2.5–2.8)	560 (30.2)	4.3	3,466 (35.3)	24.1	5.6 (5.1–6.1)
No	3,548 (66.9)	27.3	8,945 (63.7)	62.2	2.3 (2.2–2.4)	1,294 (69.8)	9.9	6,344 (64.7)	44.1	4.4 (4.2–4.7)

Note: CI = Confidence Interval, CTAS = Canadian Triage and Acuity Scale, LHIN = Local Integrated Health Network

**Fig 2:**
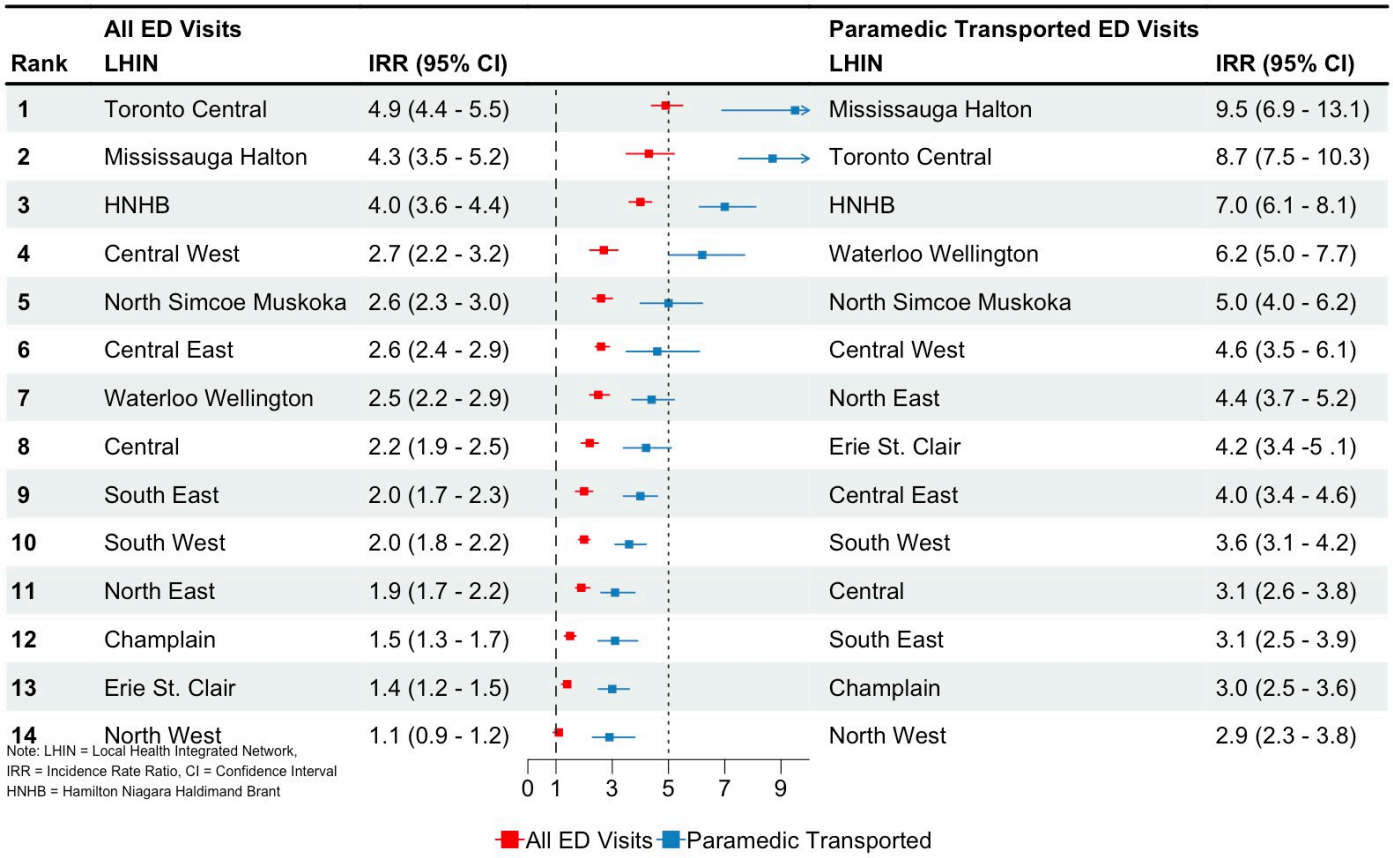
Ranked highest to lowest population-adjusted incidence rate ratios of opioid-related ED visits and paramedic transports in Ontario by Local Health Integrated Network boundaries.

Fig 2 shows ranked IRRs across geographic locations for opioid-related ED visits in a comparison between 2009 and 2019. Population-adjusted rates of ED visits and paramedic transported visits increases across all LHIN’s. For ED visits, Toronto Central (IRR 4.9, 95% CI 4.4–5.5), Mississauga Halton (IRR 4.3, 95% CI 3.5–5.2) and Hamilton Niagara Haldimand Brant (IRR 4.0, 95% CI 3.6–4.4) had the largest increases of population-adjusted annual incidence. Of paramedic transported ED visits, the largest rates were observed in LHINs Mississauga Halton (IRR 9.5, 95% CI 6.9–13.1), Toronto Central (IRR 8.7, 95% CI 7.5–10.3) and Hamilton Niagara Haldimand Brant (IRR 7.0, 95% CI 6.1–8.1).

## Discussion

Opioid-related ED visits grew substantially over the decade preceding the COVID-19 pandemic, specifically 2016 and 2017. Incident rates of paramedic transports doubled, becoming the primary mode of transport to the ED. Opioid-related visits became more clinically severe, though the overall rates of ED discharges remained consistent. After adjusting for population growth, opioid-related ED visits grew throughout all of Ontario, though considerable growth was found in more urbanized regions.

Our findings align with previous national and international literature, highlighting the substantial growth of opioid poisoning and opioid-related ED visits during 2016 and 2017 [2, 17, 18]. However, prior studies do not account for population-growth, nor do they examine the implications on paramedic systems that respond to 9-1-1 emergencies. Research suggests the growth of opioid poisoning and opioid-related ED visits during 2014 and 2015 rose to 21 per 100,000 across Ontario, a result consistent with our findings prior to the 2016 and 2017 visit spikes. The drastic increase in visits between 2016 and 2017 we determined are underreported in the literature for Canada. International findings parallel our results, citing a 29.7% increase across the United States of America over this timeframe when ED visits increased markedly [19]. Consistent with previous literature, our study found young adults (19 to 35 years) had the largest increase of ED visits and aligns with the largest age demographic that die from opioid overdoses and poisonings [2, 18–20]. Notably, minimal research describes the changes in acuity scores for opioid-related visits, and quantifiable estimates of the impact of these visits on paramedic systems are lacking in the literature.

Prescription of high-dose opioids is one potential explanation for our observation of increased opioid-related ED visits. A study of opioid dispensing in 2011 found Ontario had the largest high-dose opioid dispensing rates amongst all Canadian provinces, which we postulate continued in the years following [21]. Another explanation is the surge of illicit fentanyl, fentanyl analogues and synthetic opioid use in Canada in 2016, as evidenced by illegal drug seizures and death investigations [22]. Street derivatives of opioids became more prevalent and potent in these years, notably with carfentanil, an opioid that is 100 times more potent than fentanyl [22]. More often illicit street drugs are combined with fentanyl, leading to increased risk of overdose, addiction and death, which we observed in our study with higher triage acuity scores. In British Columbia, 68% of illicit drug deaths involved fentanyl in 2016, and increased to 83% in 2017 [23]. We hypothesize that the short half-life of opioids, increased availability of reversal agents (i.e. naloxone), more accessible to community treatment options (i.e. addictions clinics, specialized case managers) and low obligation for psychiatric assessment of acute drug intoxications mitigate the risk for discharge of recurrent opioid users and contribute to the high rate of ED discharge. We propose the large surge in paramedic transports may be attributed to heightened public awareness campaigns regarding opioid use and when to call 9-1-1, as well as understanding paramedics are equipped with reversal agents.

Our evaluation of temporal changes of ED and paramedic utilization for opioid-related conditions can support interventions that improve patient-centered care while reducing use of scarce ED services [24]. We determined that growth in opioid-related ED visits concentrated to urbanized regions and younger adults could not be attributed to population growth. Identification and correction of barriers to community and complementary opioid services should be a point of focus for these cohorts to reduce the burden of opioids on the healthcare system. Such barriers include limited evening and overnight care access and poor accessibility to complementary psychotherapy for opioid use disorders [25].

Though we found an increase in medical acuity from 2009 to 2019, we did not observe a corresponding increase in the proportion of ED patients admitted to hospital or a significant increase in returned ED visit within 30 days. This is an important finding, suggesting that EDs are capable of managing and treating the vast majority of opioid-related patient conditions. We postulate the substantial spike in paramedic transports followed by high rates of ED discharge calls for exploration of equivalent or superior care alternatives in paramedic systems. Alternative paramedic care models could include transport of patients with opioid-related conditions to specific substance abuse outpatient centres that specialize in opioid addiction, dependence and mental health treatment programs [26]. Community paramedicine is a growing field of paramedicine that can provide community care and monitoring for certain carefully selected patient cohorts [27]. Commissioning community paramedics to monitor and provide specialized care for opioid users could be a feasible and proactive solution to reduce 9-1-1 calls and transports due to opioid-related disorders. Lastly, paramedics could cultivate unique specialized response units that have extended training in substance use, opioids and mental health crises to respond specifically to opioid-related 9-1-1 calls and provide chronic therapeutics in the community, such as buprenorphine with naloxone or derivatives of buprenorphine [28].

To our knowledge, this is the first comprehensive study that evaluated changes in annual incidence of ED visits and paramedic transports for patients with opioid-related conditions in Canada. Our findings are likely generalizable to other Canadian provinces and countries where reports of an opioid crisis have been reported, and where paramedic systems are similarly organized. Interventions are needed to improve management of opioid-related conditions and reduce the burden of opioids on overstrained EDs. Examination of incidence changes during the COVID-19 pandemic is needed to quantify healthcare utilization sought by patients with opioid-related conditions, and understand trends to prepare healthcare systems for the future. Lastly, it is important to note that our study did not fully capture the impact of the opioid crisis on paramedic services, as we were unable to account for paramedic responses that did not result in ED transport. This aspect presents an avenue for further research, and could include data features not captured in the ED such as quantity of 911 calls for opioid-related conditions, rationale for declining ED transport, opioid-related condition of non-transported patients and response time metrics for patients that sought paramedic care.

### Limitations

Due to the nature of retrospective observational studies, the specific cause of the surge in ED visits could not be determined. Overall paramedic utilization is underreported in this study; instances where paramedics attended to patients but ED transport was refused are not captured in this cohort. Finally, opioid-related ED visits that were coded in NACRS using heterogeneous codification that included other non-opioid drugs or alcohol could not be included to avoid bias and an overestimation of opioid-related visits. Thus, our totals of ED visits related to opioids are underestimated and conservative with regard to the true magnitude of the opioid-related crisis. A plausible explanation of our findings is physicians became more precise in their codification of visits (i.e. specifying opioids, instead of generalized terms such as ‘drug use’). We argue this is not the cause, as generalized drug codes remain consistent over the study years and did not decrease when opioid-related condition codes increased.

## Conclusion

ED visits due to opioid-related conditions increased by 165% between 2009 to 2019. The related growth in visits transported by paramedics was markedly high at 429% over the same time period. These results can inform public health stakeholders of the magnitude and one cause of growth in ED visits, and support strategies to reduce opioid-related ED visits. The preponderance of patients are discharged back to the community, which may support paramedic care models that reduce transports to EDs by providing acute and chronic therapy onsite without transport, or transporting these patients directly to specialized substance abuse centres instead of overcrowded EDs.

## Supporting information

S1 Table ICD-10 codification. ICD-10 codification of the cohort of opioid-related emergency department visits in the National Ambulatory Care Reporting System from January 1, 2009 to December 31, 2019.
(DOCX)

S1 Checklist The RECORD statement—Checklist of items, extended from the STROBE statement, that should be reported in observational studies using routinely collected health data. (DOCX)
