# Supplementary material for: Quantifying the escalating impact of paramedic transported emergency department visits for opioid-related conditions in Ontario, Canada: A population-based cohort study
Source: PLoS One. 2023 Sep 8;18(9):e0291194. doi: 10.1371/journal.pone.0291194 (PMC10490960; doi:10.1371/journal.pone.0291194)
Supplement: S1 Table — ICD-10 codification of the cohort of opioid-related emergency department visits in the National Ambulatory Care Reporting System from January 1, 2009 to December 31, 2019. (DOCX) [file pone.0291194.s001.docx]

**SUPPORTING INFORMATION**

**S1 Table. ICD-10 codification.** ICD-10 codification of the cohort of opioid-related emergency department visits in the National Ambulatory Care Reporting System from January 1, 2009 to December 31, 2019.

| **ICD-10 Code** | **Description** |
| --- | --- |
| F11 | Mental and behavioural disorders due to use of opioids |
| F110 | Mental and behavioural disorders due to use of opioids, acute intoxication |
| F111 | Mental and behavioural disorders due to use of opioids, harmful use |
| F112 | Mental and behavioural disorders due to use of opioids, dependence syndrome |
| F113 | Mental and behavioural disorders due to use of opioids, withdrawal state |
| F114 | Mental and behavioural disorders due to use of opioids, withdrawal state with delirium |
| F115 | Mental and behavioural disorders due to use of opioids, psychotic disorder |
| F116 | Mental and behavioural disorders due to use of opioids, amnesic syndrome |
| F117 | Mental and behavioural disorders due to use of opioids, residual and late-onset psychotic disorder |
| F118 | Mental and behavioural disorders due to use of opioids, other mental and behavioural disorders |
| F119 | Mental and behavioural disorders due to use of opioids, unspecified mental and behavioural disorders |
| T40 | Poisoning by narcotic and psychodysleptics |
| T400 | Poisoning by opium |
| T401 | Poisoning by heroin |
| T402 | Poisoning by other opioids |
| T4021 | Poisoning by morphine |
| T4022 | Poisoning by hydromorphone |
| T4023 | Poisoning by oxycodone |
| T4028 | Poisoning by other opioids, not elsewhere classified |
| T403 | Poisoning by methadone |
| T404 | Poisoning by other synthetic narcotics |
| T4040 | Poisoning by fentanyl and derivatives |
| T4041 | Poisoning by tramadol |
| T4048 | Poisoning by other synthetic narcotics, not elsewhere classified |
| T406 | Poisoning by other and unspecified narcotics |
| R781 | Finding of opiate drug in blood |
